# An Essay on the Origin and Nature of Tuberculous and Cancerous Diseases

**Published:** 1837-04

**Authors:** 


### Art. V.

*An Essay on the Origin and Nature of Tuberculous and Cancerous Diseases. Read to the Medical Section of the British Association, on the 23d of August*, 1836. By Richard Carmichael, m.r.i.a. &c.—*Dublin*, 1836. 8vo. pp. 56.

The facilities now afforded for the diffusion of every kind of medical information, whether good or bad, new or old,—whether the matter is intended for the benefit of the profession or of the public, of the individual who writes or sells it,—should operate as a powerful stimulus to all medical societies and associations, to exercise, in their judicial capacities, the strictest supervision over the claims of those who profess to enlighten their members, and, under their authority, the profession generally. Indeed, we are not aware of any duty of public bodies more important than that which imposes on such of them whose object is the advancement of medical science, the severe but just scrutiny of opinions or doctrines, either new in themselves, or contrary to those which are generally received as established on the principles of the best applied inductive philosophy. Every contribution brought forward, either to increase the number of established facts or to illustrate any particular doctrine in medicine, should, when its intrinsic value shall have been estimated, but not till then, receive their approbation, and be considered worthy of being recorded in the archives of the science.

Among the numerous subjects daily brought forward by the industry and zeal of medical men, there are some which are of such a nature that very little precise knowledge can be obtained respecting them. They embrace ultimate facts, around or beyond which our philosophy wanders but does not reach them. There are others, still more frequently submitted to our understandings, which assume as obscure and unintelligible an aspect as the former, from the incomplete, inaccurate, and unphilosophical manner in which their phenomena have been observed, and the false inductions sought to be established on such premises.

Under this head we feel ourselves compelled to include the subjects of the paper whose title precedes these general remarks. Although we have been long acquainted with the pathological doctrines advocated by Mr. Carmichael on the "origin and nature of tuberculous and cancerous diseases," and have perused the present illustration of them, we confess that we feel as if he had rather added to than aided in removing the obscurity which we were happy in the prospect of believing was becoming daily less and less, through the scientific investigations of other pathologists on these diseases.

To those who have had opportunities of studying pathological anatomy, more especially the heterologous formations, which comprehend the tuberculous and cancerous, a very few remarks will suffice to point out the fallacy of the doctrines maintained by Mr. Carmichael; and this we shall do by showing, 1st, that our author's researches are incomplete and inaccurate, and, consequently, that his doctrines are untenable; and, 2dly, that he confounds together diseases of an entirely different nature, and therefore has thrown much confusion over the pathology of the diseases which he has attempted to illustrate.

The present advanced state of pathological anatomy, the progress of which has been so rapid and fruitful of late years, requires of him who advances a new theory founded on the doctrines of this branch of medicine, to furnish all the evidence derivable from this source, at least, in support of it. That Mr. Carmichael has not done this in support of his views on the origin and nature of *tubercle*, is too obvious. His description of tubercle appears to have been almost exclusively taken from the phenomena which he supposes it to present in a *single* organ, viz. the lungs. Our author certainly does speak of tubercle in other organs and regions of the body, but he gives us no description of this morbid product in situations in which its physical characters are not obscured by the minute structure of the parts, and the intermixture of tissues which do not belong to it. He has committed here the same error which has led so many pathologists astray on this subject, and created for so long a period so much uncertainty and discrepancy of opinion on the nature of tuberculous matter. It was from this cause chiefly that Laennec dignified this substance with the name of *tissue.* But the more accurate and extended researches of Cruveilhier, Andral, Lobstein, &c. threw strong doubts on the legitimate application of this term to tuberculous matter; and those of Dr. Carswell have clearly and satisfactorily demonstrated that it is an amorphous product, essentially composed of an unorganizable substance, consequently having no form but that which is impressed upon it by the influence of the mechanical and physical conditions under which it happens to be placed. The descriptions and delineations of this pathologist, given in his work on the "Elementary Forms of Disease," of the tuberculous matter in different organs, in order to show the modifying influence of these conditions, have fully convinced us, and we are sure every impartial and scientific enquirer, that the name of *tissue*, *entozoon*, or any other appellation implying organization and life in this substance, involves an hypothesis which has neither fact nor analogy in its favour. We feel assured that if Mr. Carmichael will forget what he has imagined he has learned from his examination of tubercles of the lungs, and look at this morbid product in the pelvis of the kidneys, ureters, fallopian tubes, cavity of the uterus, and other hollow organs; or in artificial cavities, such as those formed in bone, in lumbar abscess, or in lymphatic glands, in all of which situations this substance occurs in masses of various extent, and presenting all the characters which pathologists regard as constituting the *type* of this product, he will agree with us that he has hitherto been strangely deceived; for he will perceive here nothing like "semi-transparent grey vesicles," or "regular compact masses like grains of shot." He will only perceive a cheesy-looking substance of various consistence, having no *elementary* form, even when examined in the microscope. It is merely in juxtaposition with the surface which had secreted it, or lies detached from it like so much foreign matter or refuse, which the lining tissues could not appropriate for the purposes of nutrition. We agree with Mr. Carmichael, that tubercles "have no connexion by means of vessels with the surrounding tissues," because the facts just stated prove this beyond the shadow of a doubt. But the same facts prove, as we have already said, something more; for, as every morbid product must, as regards its distinctive characters, be essentially the same in every organ in which it occurs, tuberculous matter in the lungs must be the same in nature as that in any of the other organs to which we have alluded; and, since in these it presents no *elementary* form, no *texture*, or *vascular* relationship, the legitimate induction would be, were the fact not already proved by actual observation, that it can form no exception to the rule in the former organs.

We feel no disposition to follow Mr. Carmichael in his speculation on the Entozoa, in order to substantiate the claims of tubercle to a place among these animals; but we cannot avoid remarking that he has much less philosophy on his side than those who have maintained the hydatic and cystic origin of the heterologous formations. Mr. C. commits, we think, a very serious mistake in not making a distinction between two things, the one possessing *vital properties*, and the other a *distinct* or *separate animal existence*. Had he claimed for tuberculous matter the possession, in a low degree, of some vital property, it might perhaps have been considered as only a matter of words; but, when he insists on its being in possession of an "independent vitality," that it is a "parasitic animal, vesicular, hollow, or opaque," (for he is not clear on this point, or rather thinks it immaterial whether the animal be *hollow* or *solid!*) we confess there is a singular lack of proof; so much so, that we are sure no zoologist would ever think of admitting any such contributions to enlarge the boundaries of his already vast domain of animal existences, even although Mr. Carmichael very humbly asks only for the "last link of the chain in the last class of animals."

But we have, perhaps, gone too far without having laid before our readers Mr. Carmichael's definition of tubercles, and his description of their anatomical characters: the following is the definition:

"In one word, it is my opinion," Mr. Carmichael says, "that they are beings possessing a vitality independent of the animal in which they are lodged, except in so far as that animal affords them, 1st, the organic particles of which they are formed, and, 2d, the nutriment which they imbibe by their own innate powers."

The following is the description of their anatomical characters:

"Tubercles, as I have stated already, have no connexion by means of vessels with the surrounding tissues in which they are imbedded; they are commonly found in small regular circumscribed masses. They at first, generally before they undergo any transmutation, have the appearance of semi-transparent vesicles; even those that are opaque, on a close examination, I have often found to be hollow; in fact, they are thickened vesicles or cysts." (P. 21.)

We may observe here, that what has been hitherto considered and described by pathologists as tuberculous matter, is, according to our author, "the dead substance of the dead and softened" vesicle, cyst, or entozoon. Now, although we think that we have already shown the fallacy of the definition and description of the anatomical characters of tubercles, such as we have quoted it from Mr. C.'s essay, we cannot avoid stating that his definition is a mere assumption, which can derive no countenance from the anatomical characters of the supposed animal to which it is applied. He does not even afford us the satisfaction of telling us what means he employed to ascertain their anatomical characters. Indeed, he is not, as we have already said, very clear on this point; for he talks of them being semi-transparent vesicles or opaque vesicles, and concludes by saying that they are in fact thickened vesicles or cysts; nay, he is determined to have them animals, whatever appearance they present, even should they resemble grains of shot; for he adds, by way, we presume, of removing every possible objection, "I do not see why their solidity should be an objection to their possession of an independent vitality." It need hardly be observed, that such dubious language and such loose reasoning is unworthy of serious consideration. A pathologist claims the attributes of vitality and an independent existence for a thing which he cannot bring before our senses under any definite form! or, rather, which he represents as possessing no essential elementary *material* character!

But we have dwelt too long on this part of our subject, and shall cast a glance at other evidence, which Mr. Carmichael conceives sufficient to prove the possession of vitality in tubercles, viz.—1st. "they preserve themselves from those changes to which all dead or inanimate animal matter immediately enters when subjected to moisture and a temperature above the freezing point;" and, 2d, "as long as they themselves retain life, they do not occasion any stimulus or disturbance in the parts in which they have their nidus, to throw them off." Now, we shall only briefly observe, that the first part of the evidence brought forward by Mr. Carmichael in proof of the vitality of tubercles, is a gratuitous assumption, and besides, granting, for the sake of argument, that tubercles do not undergo decomposition for a long period (say for many months), this of itself would be no proof of their being in possession of vital properties and life. *Dead* animal matter, when subjected to the influence of moisture and a temperature above the freezing point, does not undergo decomposition, or any other disorganizing process, so rapidly as Mr. Carmichael supposes. Masses of inspissated mucus, albumen, and fibrine are long retained in cysts without suffering such a change; and soups, fish, and meat of various kinds, are preserved in hermetically sealed vessels for a long period, subjected to moisture even in warm climates, without undergoing any very perceptible alteration. Hence tubercles may be unorganized or inorganic substances, and yet be so situated as to suffer no alteration from such agents. With regard to the second part of the evidence in support of the vitality of tubercles, it may also be characterized as an assumption, without a single proof in support of it. We might assert the contrary,—viz. that tubercles do irritate and disturb the parts in which they have their nidus,—with equal claims to be believed. But this is not the point at issue. It is contended that they do not, so long as they *retain life*, occasion any stimulus or disturbance. Now, we have been accustomed to believe that the possession of life was the most essential means towards the doing of mischief among animals, as well as among men. All we know of intestinal worms has taught us and our patients the reverse of Mr. C.'s opinion; and the shepherd would not be persuaded that his sheep stagger and die because of the death of the entozoa which he knows are lodged in their brain. We have been informed of death succeeding to repeated convulsions produced by the presence of an ascaris lumbricoides in the ductus communis choledochus of a boy, and of a large cysticercus cellulosa in one of the lateral ventricles of the brain of a female. But, because entozoa, and morbid formations of various kinds, may be found in the body without our even having been able to suspect their existence during life, are we to conclude that they produced no morbid excitement, no modification of innervation, secretion, or nutrition? most assuredly not. Do we not meet with marked traces of the remains of pericarditis, peritonitis, pleuritis, with chronic abscess, caries of bone, nay, tubercles in different organs, even in the *dead state*, in individuals in whom the usual symptoms were either absent or so imperceptible as to escape our most careful scrutiny? Under these circumstances it is hardly necessary to observe that the production of irritation can be no test of the presence or absence of vitality. It occurs in living tissues submitted to the contact of bodies or substances, organized or inorganic, under a variety of circumstances which we cannot appreciate; and, as it often escapes our cognizance when it does occur, it would be equally insubstantial and delusive to employ it as a means of determining a character so important as that sought for by our author.

There are several points in Mr. Carmichael's essay in reference to the formation of tubercles which we had intended to notice; but, as we have already exceeded the limits which we had originally assigned to these observations, we shall only allude to one or two mistakes which he has committed by a misconception of the authors whose opinions he has criticised. In order to support his own theory, Mr. Carmichael endeavours to show that the opinions of Drs. Carswell and Clark are at variance with their own statements, regarding the nature of what are commonly called scrofula and tubercular phthisis. Whoever has read the admirable works of these two authors on tubercular diseases, viz. that on "Tubercle" of the former, and that on "Pulmonary Consumption, &c." of the latter author, cannot have failed to perceive that the terms *tubercular cachexia* and *tubercular diathesis* are employed generically, to embrace a group of diseases *originating* in a peculiar morbid condition of the economy, either congenital or acquired, the specific anatomical character of which is the formation and presence of an unorganizable substance, in various organs of the body, named tubercle. Phthisis pulmonalis, tabes mesenterica, scrofula, struma, are the diseases comprehended under this generic term, because all of them originate in the same general morbid condition of the economy, and because in all of them the same morbid anatomical product is constantly found at a certain stage or period of their progress. They constitute species and varieties only in so far as the organs, systems, tissues, &c. affected, differ in different cases or individuals. We cannot comprehend Mr. Carmichael when he says that Drs. Carswell, Clark, and Todd confound *scrofula* and *tubercle*, and that they argue for the identity of the two; for the two former of these authors, as we have already shewn, do no such thing. They do not maintain that *scrofula* is identical with *tubercle;* for scrofula implies the disease; tubercle, its substantive product. Scrofula, or the tubercular diathesis, may exist without the presence of the latter. Hence we speak of scrofulous glands as indices or manifestations of the tubercular diathesis, but not as tubercles; although actual anatomical observation has shown that such glands, either *before* they have suffered much enlargement or inflammation, or *after* they have been for an indefinite period the seat of chronic inflammation, always contain a greater or less quantity of tuberculous matter.

The only other misinterpretation on the part of Mr. Carmichael to which we shall allude, is in reference to the influence of inflammation in the production of tubercle. "How will Dr. Carswell reconcile," says Mr. Carmichael, "the two following passages?* at page 267 we read as follows 'Under such circumstances it would be absurd to ascribe the origin of tuberculous matter to inflammation;' and after arguing, (continues Mr. Carmichael,) the identity of scrofula and tuberculous diseases, we find, at page 259, the following observations:—'inflammation of any organ may be followed by the deposition of tuberculous matter in that organ in the manner in which we have already explained. We have frequent examples of the subcutaneous glands of the neck, and submaxillary

* Encyclopædia, [Cyclopædia of Pract. Med.] vol. iv.

glands, becoming tuberculous after an acute attack of inflammation, although previously neither enlarged, indurated, nor otherwise diseased." We are surprised that Mr. Carmichael should call upon Dr. Carswell to reconcile statements the meaning of which is so obvious, and so consonant with the doctrines which this pathologist entertains on the nature and origin of tubercles. Dr. Carswell has, we conceive, throughout all his writings on this subject, consistently maintained, and satisfactorily demonstrated, that it would be absurd to ascribe the origin of tuberculous matter to inflammation; whilst, at the same time, he has no less satisfactorily explained, and, we think, beautifully illustrated, the manner in which this pathological state operates locally, as a *remote* or *exciting* cause of tubercular depositions. Believing, as Dr. Carswell does, that tuberculous matter is separated by a process of secretion from the blood, he considers that the *localization* of the disease, or *presence* of this substance in a particular organ, or portion of an organ, is not only frequently determined by the supervention of inflammation, but would not, in many cases, take place, had this local morbid condition not occurred. Hence he does not admit that inflammation is necessary to the formation and deposition of tuberculous matter, but that, as it gives rise to an accumulation of blood, and consequently to an increase of secretion, in the part which it affects, it is frequently followed by the deposition of tuberculous matter; that change of the constitution, termed tubercular diathesis, *previously existing*, without the modifying influence of which, the products of inflammation would have been those by which it is characterized in other or ordinary circumstances, viz. coagulable lymph, or pus.

We have now briefly to advert to that portion of Mr. Carmichael's essay in which, as we have stated, he confounds together diseases of an entirely different nature. We allude to his disquisition on the nature and origin of carcinomatous diseases, which he numbers in the same family with the tuberculous, because he has long since also classed them amongst the entozoa. Our remarks on the opinions of our author as to the claims of tubercle to such a rank in the scale of created beings, must serve as an apology for our not entering again upon this subject: for, although there are several forms of carcinoma which possess an organized structure, and might therefore give an air of probability to some such theory, nobody, who wishes to distinguish one thing from another by a name expressive of a specific difference in their relations to each other, will ever think of calling carcinoma by any name, or attaching to it any idea signifying an animal existence or member of the family of entozoa. We shall just quote Mr. Carmichael's description of carcinoma as a specimen of the extent and accuracy of his knowledge of what pathologists call the *anatomical characters* of a disease.

"There is in every carcinomatous structure two distinct substances. One is a hard cartilaginous mass, which admits of being injected; the other is of the consistence of brain, or a medullary substance, which does *not* admit of being injected. The latter I esteem the true entozoa, or parasite; the former, i. e. the cartilaginous part, I look upon as the barrier which the surrounding tissues throw up to insolate the parasite."

We should like to know what such pathologists as Andral, Cruveilhier, Lobstein, and Carswell would say of this description of carcinoma. We do not pretend to possess a minute knowledge of the anatomy of the disease, but we have examined it sufficiently often to feel ourselves justified in saying that we have never before seen such an imaginary and erroneous representation of it in print. We admit that carcinoma is composed of two distinct substances, an analogous and a heterologous; the former consisting of cellular, cellulo-fibrous or fibrous tissue, the other of the peculiar substance of the carcinomatous formation: and that these exist in various proportions, and under a variety of modifications of their physical and physiological properties: but when we are told that, contrary to the evidence of occular demonstration, again and again confirmed, one of these substances which Mr. Carmichael chooses to call cartilage is organized, because it admits of being injected, and that the other, or medullary, is not organized, because it does *not* admit of being injected, we must not only object to the accuracy of such statements, but authoritatively pronounce them to be the very reverse of the fact. For, *cæteris paribus*, of the two substances which enter into the composition of a carcinomatous tumour, the medullary possesses the greatest number of blood-vessels. So rich in blood-vessel is this substance occasionally, that it is almost as red as blood; and this not from hemorrhage, but from the presence of the aggregated multitudes of the fine capillary vessels which constitute a part of its organization. Medullary sarcoma, or fungus hæmatodes, is as obviously organized as the brain or cerebral substance which it resembles. The vascular element of the one, like that of the other, is maintained and accompanied by fine cellular or filamentous tissue, from which the peculiar substance of each may be removed by ablution, and the degree and extent of their vascular organization more accurately estimated. The pathologists whose names we have already so frequently quoted all agree in this important fact regarding the structure of the forms of carcinoma to which we have alluded; and, indeed, no one who has ever practically examined this point can entertain any other opinion. With regard to the vascularity of the *cartilaginous* substance (which we beg to notice as a discovery of Mr. Carmichael's), which admits of being injected, we leave it for the consideration of anatomists, who have hitherto found this, we believe, more than they could accomplish.

We must now take our leave of Mr. Carmichael's essay; and, if the tone in which we have treated it should appear to carry with it more than merited censure, we seek no other grounds of justification than those we have already offered, viz. the importance of the subjects, the novelty of the views, and the incomplete, inaccurate, and unphilosophic manner in which they have been investigated, and offered for the instruction of the profession. The author's rank and standing in the profession, and his acknowledged reputation as a surgeon, give an importance to his views and opinions, whether for good or evil, which claims the attention of the guardians of the press. It is for this reason that we have bestowed so much notice on this pamphlet; looking to the eminence of the author rather than the value of the work.

